# Exploring cybersexuality among adolescents: findings from a cross-sectional study

**DOI:** 10.1192/j.eurpsy.2026.10858

**Published:** 2026-07-31

**Authors:** Z. Mallek, M. Trigui, E. Mziou, M. Baklouti, Y. Mejdoub, F. Ben Dhaou, J. Jdidi, S. Yaich, M. Kassis

**Affiliations:** 1 Hygiene, Habib Bourguiba University Hospital; 2 Community Health and Epidemiology, Hedi Chaker University Hospital; 3 Social Medicine, Faculty of medicine, Sfax, Tunisia

## Abstract

**Introduction:**

The increasing use of social media and digital platforms exposes adolescents to sexual content, which may impact their psychological well-being and psychosocial development. However, data on the prevalence of cybersexuality and its associated factors remain limited.

**Objectives:**

Our study aimed to evaluate the prevalence of cybersexuality among Tunisian adolescents, assess their exposure to online pornographic content, and identify sociodemographic factors related to their cybersexual behaviors.

**Methods:**

We conducted a cross-sectional study among middle and high school adolescents in Tunisia from September to October 2024. The survey involved a self-administered, anonymous questionnaire. Cybersexuality was assessed through questions about using online platforms to discuss sexual topics or relationships and the frequency of these interactions. Informed consent was obtained from the students’ legal guardians, ensuring data confidentiality.

**Results:**

A total of 1,123 adolescents participated, of whom 39.2% (n=440) were males. Among them, 53% (n=595) were high school students. We observed that 54.5% (n = 593) reported encountering pornographic content, with 35.3% (n = 384) stating that this exposure was unintentional. The prevalence of cybersexuality among adolescents was 22.1% (n = 241, 95% Confidence Interval (CI) = [19.7-24.7]). The median frequency of pornographic platform use was 4 hours per month (Interquartile Range (IQR) = [1-13.5] hours). The analysis of factors associated with cybersexuality among adolescents revealed that it was more common among male students (Odds Ratio (OR) = 4.52; p < 0.001) and high school students (OR = 1.4; p = 0.02).

**Conclusions:**

Our study showed that cybersexuality is a growing behavior among adolescents, emphasizing the importance of adult supervision, the development of trustworthy educational resources, and the implementation of comprehensive sexual health education programs.

**Disclosure of Interest:**

None Declared

